# The Student’s Chart of the Sympathetic Nerve

**Published:** 1871-08

**Authors:** 


*The Student's Chart of the Sympathetic Nerve.* By RALPH M. TOWNSEND, M. D., Assistant Demonstator of Anatomy in the Jefferson Medical College.

This is a convenient hand map, giving a good general idea of the distribution and relations of the different branches of the sympathetic nerve. To distinguish the different nerves, the author has colored the motar nerves blue, the sympathetic yellow, and the sensory red. We should think it very useful to the student.

